# Application of machine learning classifiers for microcomputed tomography data assessment of mouse bone microarchitecture

**DOI:** 10.1016/j.mex.2021.101497

**Published:** 2021-08-24

**Authors:** Jennifer C. Coulombe, Zachary K. Mullen, Maureen E. Lynch, Louis S. Stodieck, Virginia L. Ferguson

**Affiliations:** a Department of Mechanical Engineering, UCB 427, University of Colorado, Boulder, CO 80309, United States of America; b BioFrontiers Institute, UCB 596, University of Colorado, Boulder, CO 80309, United States of America; c Laboratory for Interdisciplinary Statistical Analysis / Department of Computer Science, UCB 427, University of Colorado, Boulder, CO 80309, United States of America; d Aerospace Engineering Sciences / BioServe Space Technologies, UCB 429, University of Colorado, Boulder, CO 80309, United States of America

**Keywords:** MicroCT, Mouse Models, K-means, SVM, Microgravity, Aging, Disuse

## Abstract

The current standard approach for analyzing cortical bone structure and trabecular bone microarchitecture from micro-computed tomography (microCT) is through classic parametric (e.g., ANOVA, Student's T-test) and nonparametric (e.g., Mann-Whitney U test) statistical tests and the reporting of *p*-values to indicate significance. However, on their own, these univariate assessments of significance fall prey to a number of weaknesses, including an increased chance of Type 1 error from multiple comparisons. Machine learning classification methods (e.g., unsupervised, k-means cluster analysis and supervised Support Vector Machine classification, SVM) simultaneously utilize an entire dataset comprised of many cortical structure or trabecular microarchitecture measures, thus minimizing bias and Type 1 error that are generated through multiple testing. Through simultaneous evaluation of an entire dataset, k-means and SVM thus provide a complementary approach to classic statistical analysis and enable a more robust assessment of microCT measures.

## Highlights

• Machine learning approaches allow for the simultaneous analysis to an entire microCT dataset to minimize bias and demonstrated that collective microarchitectural changes.
• K-Means clusters and Support Vector Machine classification visualization provide intuitive interpretations of the differences in bone structure and microarchitecture between groups.
• These techniques are complimentary to common statistical testing and provide additional ways of showing differences between microCT outcomes.

## Graphical abstract

**Figure:**
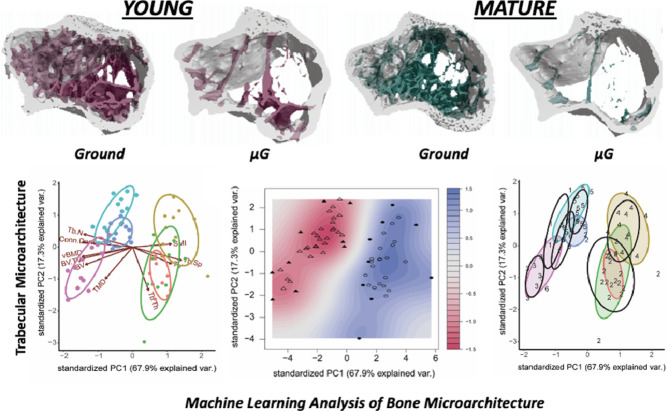

Specifications table

**Table:** 

Subject Area:	Medicine and Dentistry
More specific subject area:	Bone Biology, Orthopaedics
Method name:	*Machine Learning Classification of Microcomputed Tomography Datasets*
Name and reference of original method:	*Original Article: Submitted concurrent with manuscript to the Elsevier journal “Bone”, manuscript number BONE-D-21-00149R1* *MicroCT Outcome Measures: M. L. Bouxsein, S. K. Boyd, B. A. Christiansen, R. E. Guldberg, K. J. Jepsen, R. Muller, “J BMR Guidelines for Assessment of Bone Microstructure in Rodents Using Micro – Computed Tomography,” JBMR., vol.25(7), pp.1468–86. 2010.* *K-Means: S. P. Lloyd, “Least squares quantization in PCM”, Bell Lab, New Jersey, United States of America, Tech. Rep. RR-5497, 1957.* *J. B. MacQueen, “Some methods for classification and analysis of multivariate observations," in Proceedings of the fifth Berkeley symposium on mathematical statistics and probability. L. M. Le Cam & J. Neyman Eds. California: University of California Press, 1967. pp. 281-297.* *Rand Index: W. M. Rand, “Objective Criteria for the Evaluation of Clustering Methods,” J. Am. Stat. Assoc., vol. 66(336): pp. 846. 1971.* *SVM: A. Ben-Hur, D. Horn, H. Siegelmann, and V. N. Vapnik, "Support vector clustering" Journal of Machine Learning Research., vol. 2, pp. 125–137. 2001.*
Resource availability:	R version 4.0.0 (2020-04-24) – "Arbor Day" Copyright (C) 2020 The R Foundation for Statistical Computing Packages: prcomp, stats, ggbiplot, factoextra, e1071, kernlab

## Background

Microcomputed tomography (microCT) is ubiquitous for assessment of mouse and rat bone microarchitecture in orthopaedics and bone biology. In 2010, Bouxsein et. al., established common terminology and standardized measurements, including eighteen outcome measures describing cortical bone structure and fifteen describing trabecular bone microarchitecture [1]. Of these measures, roughly fourteen are commonly reported across both bone compartments. However, statistical approaches for the evaluation of microCT data remain simple. Our research community relies upon comparisons of individual microCT outcome measures that are commonly made using standard *p-value* based statistical analyses, such as ANOVA with Tukey's Honest Significant Difference (Tukey's HSD), Student's T-Test, and Mann-Whitney Wilcoxon tests. On their own, these assessments of significance differences in microCT outcome measures fall prey to the following critiques/weaknesses:

1) The standard of ɑ < 0.05 is arbitrary and thus may not be an appropriate metric for scientific findings. Therefore, it follows that deeming a finding “significant” is also arbitrary and should not be given undue weight in an interpretation [2,3].
2) *p-value*s are notoriously misapplied, misinterpreted, and not repeatable. *p-value*s fundamentally do not provide substantial evidence that a treatment is the cause of differences in two datasets. *p-value*s only reflect that a null-hypothesis is rejected and that an alternative hypothesis is more favorable [4,5].
3) Rodent studies often have low sample sizes due to the costs and ethical consideration that are associated with animal research. With insufficient power, a *p-value*’s interpretation is further hampered, and researchers should be more conservative in their statements given low sample sizes [6]. Reporting power and effect size is therefore critical especially when sample size is limited [6].
4) Performing multiple ANOVAs, such as performing multiple pairwise or Student's t-tests to evaluate many outcome measures increases the chance of a Type 1 error [7,8].

Further, while individual measures of bone structure and microarchitecture can describe how bone's morphology is changing (e.g. thinner trabeculae, greater cortical area), reporting multiple measurement comparisons can be overwhelming and confusing. Reporting multiple statistical significance of individual microCT measures may also suggest that each measure is of equal weight. Yet, certain measures may have greater contributions to the variance among treatment groups, and some outcome measures carry some redundancy (e.g., trabecular thickness and trabecular spacing). This can make results difficult to interpret, especially when research questions are based on a hypothesis designed to test if *a specified treatment does/does not significantly affects bone structure and microarchitecture.*

Thus, we suggest additional means of demonstrating differences between microarchitectural outcomes to enable robust assessment of the multiple outcome measures that are simultaneously observed from microCT imaging of rodent bones. Machine learning classification can be used as an initial visual inspection of microCT data to augment conventional group-wise comparison analyses or to create predictive models for future studies. Here, we outline two basic machine learning algorithms - unsupervised, k-means cluster analysis and supervised Support Vector Machine classification - to visualize microCT data and provide additional evidence of differential effects of a treatment. We provide an R Markdown file and a sample microCT dataset from two experiments where mice flew in microgravity for validation of these methods and to aid investigators in implementing these approaches in their own studies. Our companion paper [9] presents both the parametric analysis and machine learning analysis of the full dataset. PCA, k-means, and SVM are thus complimentary techniques that enable a deeper understanding of datasets such as those that utilize many outcome measures to describe microarchitectural assessment of rodent bone.

## Procedure

### Principal component analysis

While principal component analysis (PCA) of microCT outcome measures has been used to evaluate callus structure [10], models of osteoarthritis [11] and obesity [12], and mandibular trabecular bone [13], it is seldom used for bone microarchitectural assessment. If more widely applied, this approach could become a ubiquitous statistical tool for more thorough assessment of microCT data. The PCA representation of a data set is a rotation of the data set into the coordinate system that explains the greatest variance. Each principal component is an orthogonal, linear combination of the independent variables. Here, PCA was used to determine whether variation in the microarchitectural measures, many of which were interrelated, could be explained in terms of a smaller number of independent variables. This dimension reduction technique allowed us to evaluate differences in microarchitecture measures among treatment groups in two dimensions (PC1 and PC2) rather than a multidimensional space that is harder to interpret and impossible to visualize [14,15]. Furthermore, PCA reduces the data into an orthogonal form, which removes any collinearity between predictors that might make any multivariate ANOVA and linear model predictor-level results meaningless.

**Fig. 1:**
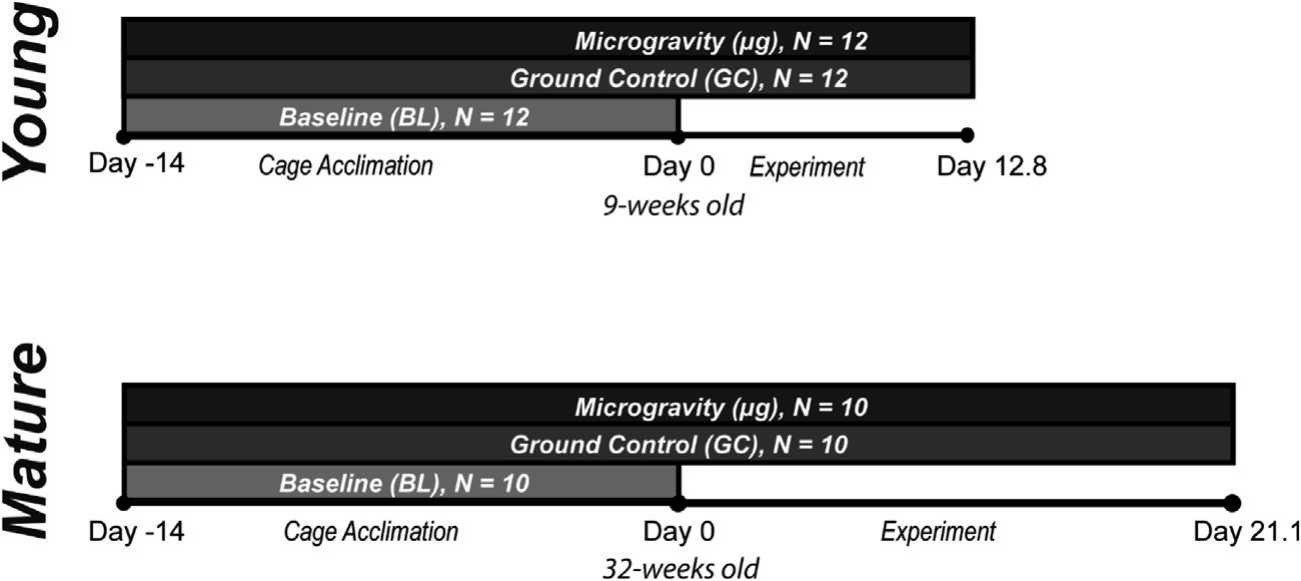
**Study design.** Six treatment groups were compared in this study: Young Baseline, Young Ground Control, Young Microgravity, Mature Baseline, Mature Ground Control and Mature Microgravity.

The accompanying data sets are of cortical structure and trabecular microarchitecture of bones from mice flown on spaceflight missions. “Young” mice (9-weeks-old at time of launch) were flown on the ∼13 day Space Transport Service (STS) 118 Space Shuttle mission. Separately, “Mature” mice (32-weeks-old at launch) were flown to the International Space Station on the SpaceX Commercial Resupply Service, CRS-4 mission for 21 days (Fig. 1**.**). The mice were the same sex (female), strain (C57BL6), and were provided with near-identical food, water, and housing. While duration in microgravity is a confounding factor, these two groups allow an assessment of how microgravity exposure differently affects growing and mature bone in female mice.

#### Construct PCA component tables

For each data set (i.e., cortical microCT outcome measures or trabecular microCT outcome measures), the percent variation and cumulative percent variation explained by each microCT measure were evaluated using the R package prcomp [16]. For each set of measures, all data were included. As the principal components, rather than the individual microCT measures themselves, were used for machine learning (ML) classification, we first assessed if a lower dimensional representation retains the information of the original data set. For example in Table 1, for the distal femur, the first principal component (PC1) explained 67.92% of the variation in our dataset, and together with the second component (PC2), 85.24% of the variation in the data is captured. In each case, PC1 and PC2 are representative of the dataset and can subsequently be used in machine learning classification modeling. The utility of PC1 and PC2 in other models can first be assessed by considering the cumulative percent variation, which is a description of how much variability of the original dataset (i.e. how colinear is the original dataset) is captured by the principal components. Consequently, higher cumulative percentages will provide a better model of the original dataset than lower values. While higher dimensions (i.e. more principal components) may increase the cumulative percentages and thus the clustering accuracy, there is a loss of matching visual clarity when including additional principal components. As such, we follow the heuristic of a minimum of 80% cumulative variance explained by the first two principal components for interpretable and reliable results of ML models. Principal components beyond PC1 and PC2 may still be useful to reduce the dimensions of the datasets.

**Table 1:** Trabecular bone microarchitecture principal component analysis (PCA)**.** For the trabecular microarchitecture dataset of the proximal tibia and distal femur the percent variation explained, cumulative percent variation explained and loadings for the first three principal components are listed. Loadings describe how much each microCT outcome measure contributes to a particular principal component. The larger the absolute value of the loading, the strong the relationship to a particular principal component. The sign of the loading indicates whether the microCT outcome measure is positively or negatively correlated with a given principal component.

	TIBIA			FEMUR		
	*PC1*	*PC2*	*PC3*	*PC1*	*PC2*	*PC3*
*% Variation Explained*	62.27	18.40	10.39	67.92	17.32	6.72
*Cumulative % Variation Explained*	62.27	80.67	91.06	67.92	85.24	91.95
*TV*	0.21	-0.09	0.73	0.29	-0.26	-0.40
*BV*	-0.37	-0.22	0.04	-0.35	-0.27	-0.21
*BV/TV*	-0.38	-0.19	-0.06	-0.37	-0.18	-0.10
*Conn.Dens*	-0.38	0.10	0.08	-0.37	0.08	0.03
*SMI*	0.13	0.51	-0.50	0.28	0.05	0.72
*Tb.N*	-0.39	0.13	-0.03	-0.36	0.20	0.18
*Tb.Th*	0.20	-0.50	-0.42	0.12	-0.68	0.08
*Tb.Sp*	0.38	-0.14	0.02	0.36	-0.21	-0.13
*vBMD*	-0.28	-0.50	-0.12	-0.37	-0.15	-0.01
*TMD*	0.31	-0.32	-0.13	-0.20	-0.50	0.46

#### Construct PCA biplots

**Fig. 2:**
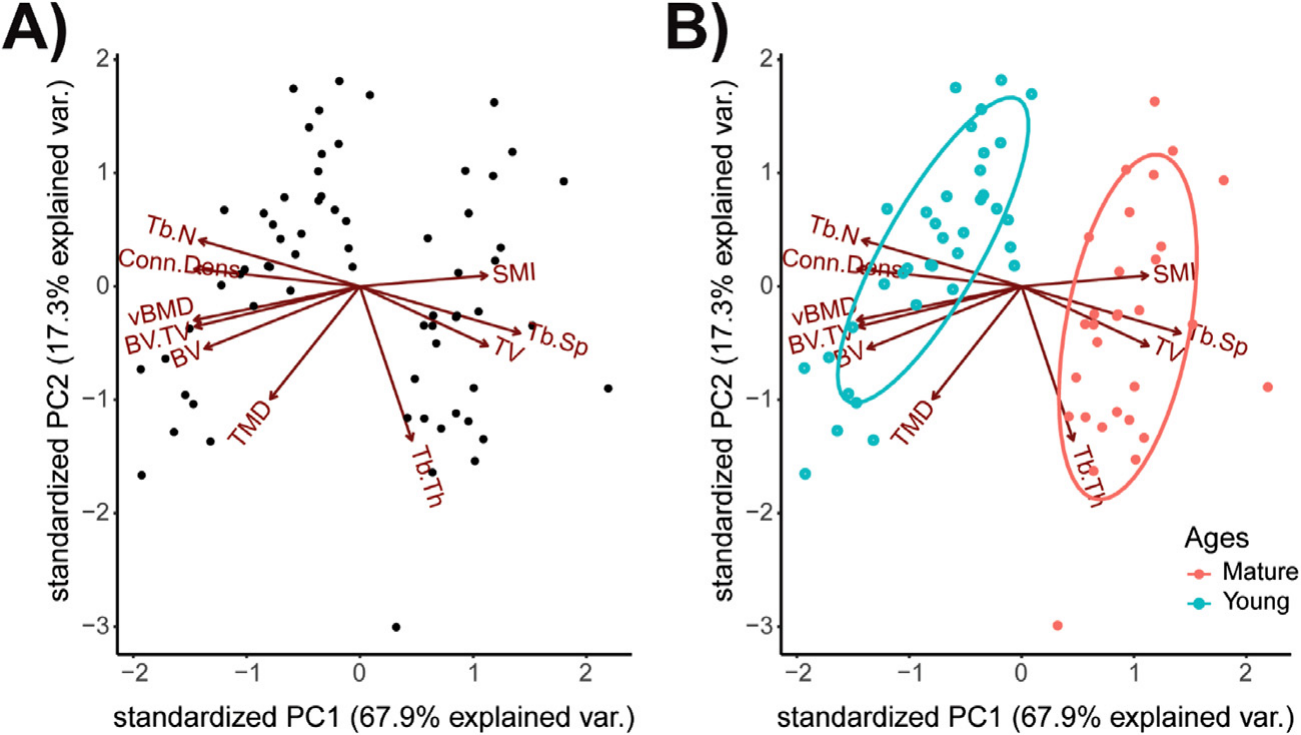
**PCA biplot of distal femur trabecular MicroCT outcome measures in young and mature mice.** A) PCA biplot of PC1 and PC2 of the distal femur trabecular dataset and B) with grouping based on mouse's age (e.g., Young or Mature). PCA biplots of trabecular microarchitecture present the inter- and intra-group variance; where each point corresponds to an individual mouse and is within a shaded 95% CI ellipse of the mouse's age group.

After principal components have been calculated, data are plotted in 2D in the PC1/PC2 plane using the R package ggbiplot [17] (Fig. 2). PCA biplot shows both PC scores of each sample (or in this study, mouse) represented by a dot and loadings of microCT variables represented by a vector (Fig. 2.A). The further away vectors are located from a PC origin, the more influence they exert on that PC. Loading plots also hint at how variables correlate with one another: i.e. a small angle implies positive correlation, a large one suggests negative correlation, and a 90° angle indicates no correlation between two characteristics. Here, PC1 is the x-axis as it comprises the most variation from the datasets, and PC2 is the y-axis as it comprises the second highest amount of variation from the datasets (see Table 1). Mice that have similar trabecular bone microarchitecture are closer together. If microCT data collected from two groups of mice are different based on, say, PC1 (Fig. 2.B), such differences are likely to be due to the microarchitecture measures that most heavily influence PC1. PC1 accounts for the most variation in the dataset, while PC2 reveals the second most variation. Therefore, differences in distance between data points along PC1 axis are larger than the similar-looking distances along PC2 axis. These plots also serve as “true groups” for comparisons with k-means clusters.

### K-means cluster analysis - unsupervised machine learning

K-means clustering is one of the simplest but highly utilized unsupervised machine learning algorithms [18]. The objective of k-means is to group similar data points together and discover underlying patterns without the use of treatment labels (e.g., “Mature”, “Young”, “Baseline”, “Ground Control” or “Microgravity” in the accompanying dataset). Visualization of the resulting clusters can provide an initial intuitive understanding of differences between treatment groups in microarchitecture outcomes either before, or in addition to, traditional hypothesis testing. To achieve this objective, k-means looks for a fixed number (k) of clusters in a dataset, either specified *a priori* (based on the study design) or found through optimization using either the shoulder or average silhouette approach from scree plots. Differences in study design-based and optimized values of k may also provide insight into the effects of a treatment on bone microarchitecture.

#### Optimization of K-Means

**Fig. 3:**
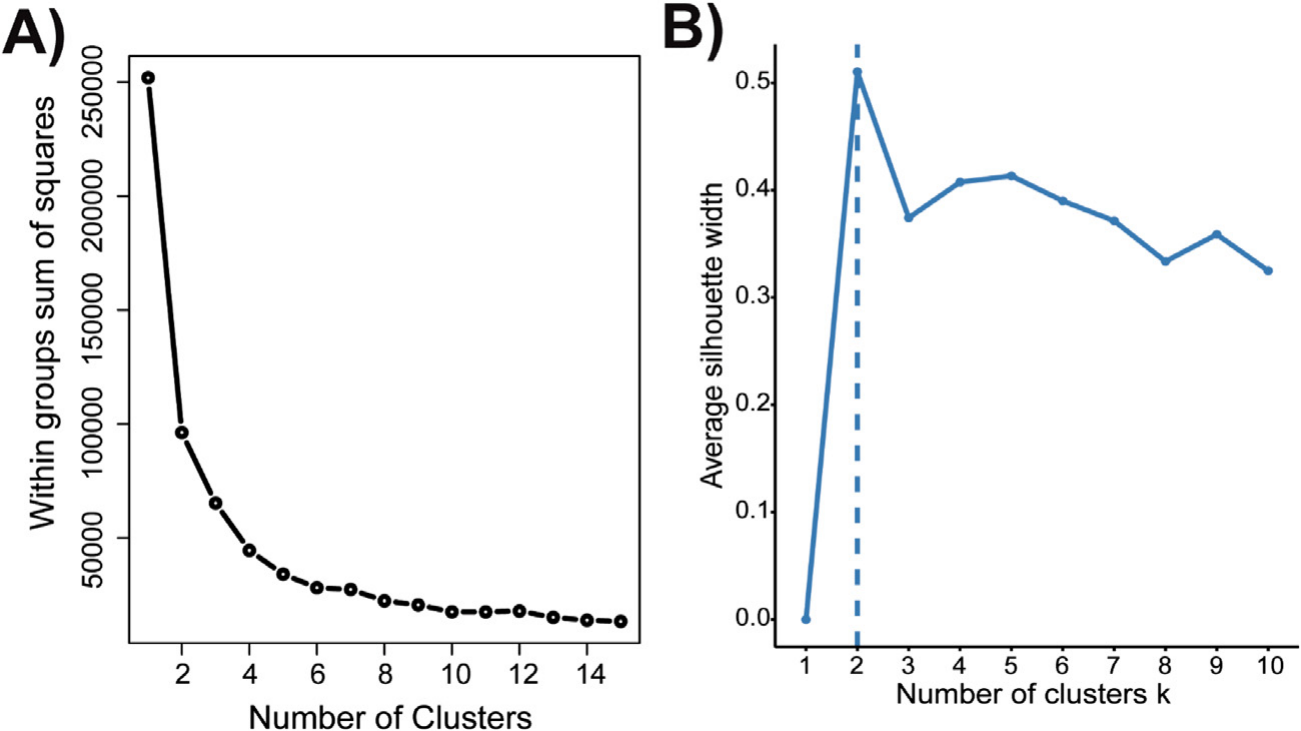
**K-Means cluster analysis scree plots for k-cluster optimization of distal femur trabecular microarchitecture PC1 and PC2.** A) Scree plot of k-means cluster number parameters n-start = 25, iter.max =1000 using the Elbow method to select number of clusters. B) K-means cluster optimization by Average Silhouette method, using factoextra [19]. Both the Elbow and Average Silhouette method determined the optimal number of clusters is k=2.

The elbow method (Fig. 3.A) interrogates the total intra-cluster variation or total within-cluster sum of square (WSS) as a function of the number of clusters. One should choose a number of clusters so that adding another cluster does not significantly improve the total WSS. The location of a bend (elbow) in the plot is generally considered as an indicator of the appropriate number of clusters (Fig. 3.A). However, the interpretation of where the “elbow” occurs via visual inspection can be highly subjective. An alternative to the elbow method is the silhouette method, which measures the quality of a clustering, or how well each data point (e.g. mouse) lies within its assigned cluster (Fig. 3.B). The average silhouette method computes the average silhouette of observations for different values of k, where a high average silhouette width indicates a good clustering. The optimal number of clusters k is the one that maximize the average silhouette over a range of possible values for k (Fig. 3.B). Therefore, the location of the maximum is the optimal number of clusters. Of interest, while k=2 cluster was determined to be optimal from both the elbow and silhouette method, k=5 would be the second optimal number of clusters as it is the second maxima in the silhouette plot (Fig. 3.B). Two clusters, (k=2) in this dataset most likely corresponds to the two different ages of the mice (Mature or Young), which can also be seen in the PCA biplot with 2 clusters. However, k=5 is a surprising outcome as there are 6 treatment groups or “true groups” from our study design (Fig. 1), and thus we would anticipate one cluster for each of these treatment group.

With k specified, the algorithm initializes centroids by first shuffling the dataset and then randomly selecting k data points for the centroids without replacement. The k-means algorithm starts with a first group of randomly selected centroids, which are used as the beginning points for every cluster. Then, iterative calculations are performed to optimize the positions of the centroids so that the dataset is partitioned into k pre-defined distinct non-overlapping subgroups (clusters). It continues to iterate until there is no change to the centroids, i.e. assignment of data points to clusters is not changing. Furthermore, optimization of intra-cluster similarities and inter-clusters differences are maximized, such that the sum of the squared distance between the data points and the cluster's centroid is at the minimum. Because k-means is sensitive to an initial randomization, any k-means package will include an argument for initializations (i.e., the starting number centroids). We found 25 to be stable for this dataset.

#### K-Means clusters by mouse age

**Fig. 4:**
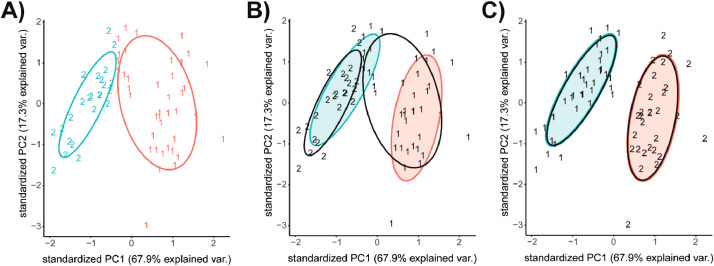
PCA biplot and k-Means K = 2 clusters of distal femur trabecular microarchitecture PC1 and PC2. A) K-means cluster analysis of distal femur trabecular PC1 and PC2; these plots demonstrate how the k-means analysis was able to predict assignment of Mature and Young mice treatment. Numbers and colors (red, #1 and blue, #2) distinguish between clusters from k-mean algorithm of PC1 and PC2 and do not directly correspond with true age groups (e.g., Young and Mature). B) Overlap of true groups and k=2 clusters where shaded ellipses (blue and red) represent the true groups from the PCA biplots (Young and Mature), and black ellipse and numbers are from k-means clusters. C) Overlap of true groups and k=2 clusters with scaled PC1 and PC2 values.(For interpretation of the references to color in this figure legend, the reader is referred to the web version of this article.)

We created an initial k-means cluster plots of PC1 and PC2 of distal femur trabecular microarchitecture outcome measures with k=2 (Fig. 4.A), as per the previous optimization. Table 2 details how many samples are in each cluster from each group. Of note, some of the Young Baseline and Young Microgravity mice were sorted into Cluster 2, which is predominantly Mature mice. This is illustrated in Fig. 4.B, where the True Groups are shaded in blue (Young) and red (Mature), the k-means clusters are overlapped in black, and cluster 1 spans both the blue and red ellipses. These misclassifications may be due to the difference in the proportion of variance explained by PC1 (67.9%) as compared to PC2 (17.3%) as they are given equal weight in the k-means analysis. The weighting of PCs by was achieved by multiplying the proportion of variance explained by each PC to its corresponding vector. For example, as the proportion of variance explained by PC1 was 67.9%, therefore the vector of PC1 was multiplied by 0.679 and similarly the vector of PC2 was multiplied by its proportion of variance, 0.173. This was achieved using the scale argument of prcomp [16]. However, when we perform the k-means analysis with our dataset weighed by the proportion of variance explained by the corresponding principal component, the classification greatly improves and the k-means matches our true groups (Fig. 4.C). In fact, all mice were correctly classified by age once the dataset is weighted by both PC1 and PC2.

**Table 2:** K-Means K = 2 clusters of distal femur trabecular microarchitecture PC1 and PC2

	Cluster 1	Cluster 2
*Mature Baseline*	0	10
*Mature Microgravity*	0	10
*Mature Ground Control*	0	10
*Young Baseline*	7	5
*Young Microgravity*	5	7
*Young Ground Control*	12	0

#### Comparison of k-Means clusters and true groups plots with rand index

For this study, we investigated k=2 as it was determined through optimization and k = 6 which was selected *a priori* to match the study design. We then compared each k-means output to the corresponding true groups.

An adjusted Rand Index was calculated for each k-means cluster analysis (i.e., k=2 and k=6) as it compared to the true clustering of the PCA plots using the R package fossil [20]. The index has a value between 0 and 1, with 0 indicating that the two data clusterings do not on any more points than random chance would give rise to, and 1 indicating that the data clusterings are exactly the same [21], [22], [23]. For example, on a data set with 3 observations, the clusterings “A, A, B” and “B, B, A” are identical groupings with an adjusted Rand Index of 1 because each is a grouping of the first two observations into the same class and the third observation into its own class. These index values suggests that the k-means clustering of the microCT data set using the first and second principal components is similar to the real “clustering” of the data. Additionally, adjusted Rand Index values may be used to compare the k-means classification strengths between groups. For example, the adjusted Rand Index value for k=2 on weighted PC1 & PC2 values of trabecular bone of the distal femur is 1, meaning all mice were correctly classified based on age (i.e., Mature or Young). By contrast, the adjusted Rand Index for the unweighted PC1 and PC2 was only 0.698, suggesting some mice were not correctly classified, and weighting by contributions of principal components improves the classification.

**Fig. 5:**
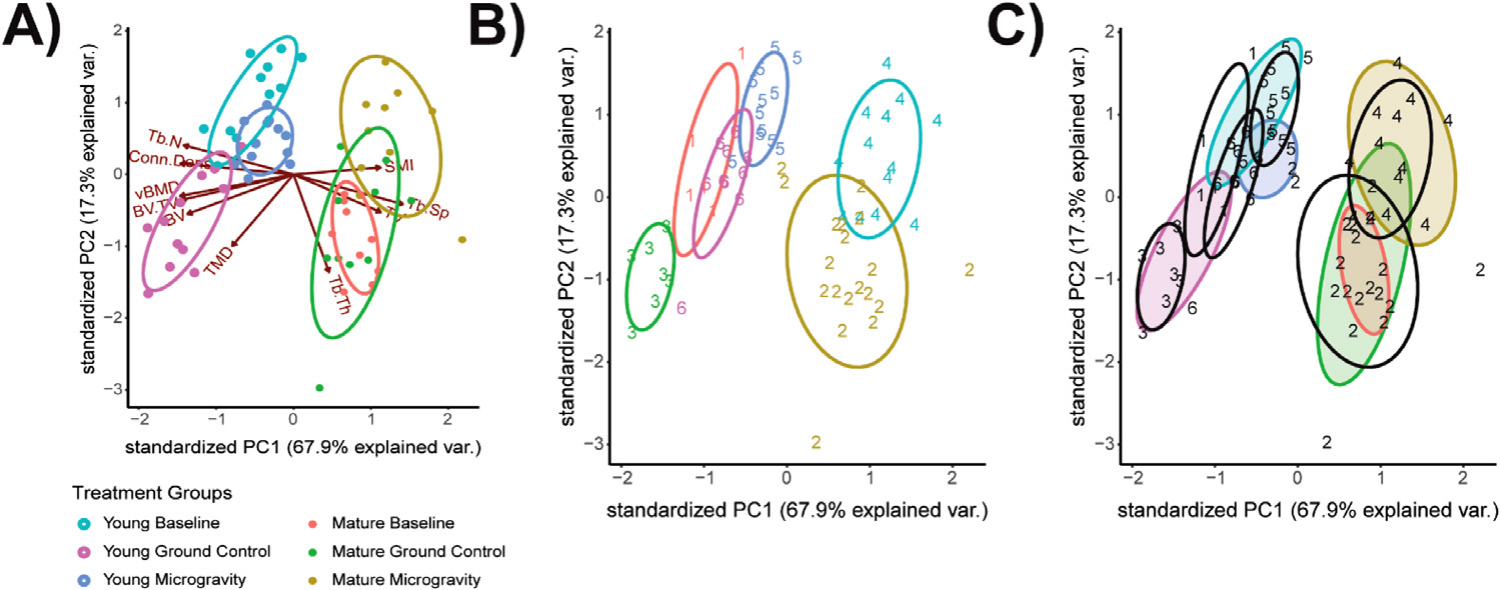
PCA Biplot and k-Means K = 6 clusters of distal femur trabecular microarchitecture PC1 and PC2. **A**) PCA biplots of PC1 and PC2 of the distal femur trabecular dataset with grouping based on mouse's age and loading (i.e., Young Baseline, Mature Microgravity, etc.). **B**) K-means cluster analysis of distal femur trabecular PC1 and PC2 where k=6. Numbers distinguish between clusters from k-mean algorithm of PC1 and PC2 and do not directly correspond with true age groups (i.e., Young Baseline, Mature Microgravity, etc.). C) Overlap of true groups and k=6 clusters where shaded ellipses represent the true groups from the PCA biplots (A), and black ellipse and numbers are from k-means clusters (B).

For our k-means model of k=6, the number of clusters has been selected *a priori* due to the study design, rather than selecting the optimal number of clusters that the algorithm would infer from the data (in our case k=2). Therefore, random clustering may have arisen from the difference in optimal number of clusters and user defined number of clusters. For example, in Fig. 5.B, the k-means algorithm only finds two clusters (clusters 2 and 4) within the Mature population of the dataset, rather than three (Fig. 5.A, Baseline, Ground Control and Microgravity). As we have selected k=6, the algorithm creates for four more clusters within the Young population of the dataset (Fig. 5.B, clusters 1, 3, 5 and 6). As there are also only three true groups for the Young mice (Fig. 5.A), one of these clusters is random. For example, the Rand Index score for k=6 is 0.794, yet the adjusted Rand Index is equal to 0.267, which is more appropriate for the number of misclassifications between true groups and k=6 clusters (Fig. 5.C). Given these differences, the adjusted Rand Index provides a more reliable measure of comparison than the unadjusted Rand Index (Table 3).

**Table 3:** K-Means K = 6 clusters of distal femur trabecular microarchitecture PC1 and PC2.

	Cluster 1	Cluster 2	Cluster 3	Cluster 4	Cluster 5	Cluster 6
*Mature Baseline*	0	0	0	2	0	8
*Mature Microgravity*	0	0	0	7	0	3
*Mature Ground Control*	0	0	0	4	0	6
*Young Baseline*	4	0	2	0	6	0
*Young Microgravity*	5	0	0	0	5	2
*Young Ground Control*	1	8	3	0	0	0

Comparison of “true groups” to the k-means clusters demonstrates that microCT assessment of trabecular bone outcomes from the distal femur Of Mature Baseline and Mature Ground Control are nearly indistinguishable. This may explain why k=5 was found to be the second optimal number of clusters. In fact, the adjusted rand index for k=5 is 0.347, higher than k=6. These results could be presented concomitant with Student T-tests, ANOVAs, and Post-Hoc analysis to provide additional evidence for a biological interpretation of differences in microCT data or where differences were absent (i.e., in the case of Mature Baseline and Mature Ground Control groups).

### Support vector machine classification - supervised machine learning

Support Vector Machines (SVMs) are another common machine learning classifier. Like k-means, SVM can be used to create an initial, intuitive visual model of differences in microCT outcome measures between treatment groups. Additionally, SVM models can provide a measure of how well treatment groups can be classified based on their microCT outcome measures, which could be reported in addition to an ANOVA or linear model. Unlike k-means analysis, SVMs are a subclass of supervised machine learning techniques as they optimally partition the data into two or more groups based on their known labels (i.e., Young Baseline, Young Ground Control, Young Microgravity, Mature Baseline, Mature Ground Control, Mature Microgravity for the accompanying dataset). An SVM is linear divisor of the data, separating observations by lines and/or planes. By using a dimension reduction technique (PCA for our data set), we can utilize SVM in 2D (PC1 and PC2 from §1) to generate a model of a dividing line that maximizes the margin between the two sets of points.

**Fig. 6:**
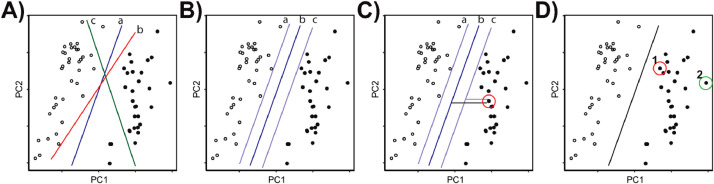
Visualization of the process of linear SVM optimization for Young vs Mature mice. A-D) represent the main steps of SVM classification. **A**) Mature mice are represented by closed circles and Young mice by open circles with lines a, b, and c as possible fit lines (representing hyperplanes) to divide the data. **B**) Multiple fit lines (a, b, c) that separates the data are compared. **C**) The best fit line (b) creates the largest margin between the separating hyperplane and the observations points. The point circled in red is called a “support vector” as it helps to determine the position and the orientation of the hyperplane. **D**) Confidence of a point belonging to a group can be calculated based on the points distance from the margin. For example, we are more confident that Point 2 (green circle) is correctly classified as a Mature mouse than Point 1 (red circle) because it is a greater distance from line of division between the two groups. (For interpretation of the references to color in this figure legend, the reader is referred to the web version of this article.)

For example, using the accompanying dataset, we assessed the classification strength of the model on separating the sets of points by mouse age (e.g., Young vs Mature). Initially, the SVM model tries to find the best line (or hyperplane) to divide the data. In Fig. 6.A, Mature mice are represented by closed circles and Young mice by open circles with lines a, b, and c as possible fit lines (representing hyperplanes) to divide the data. The best fit line not only separates the data, but also creates the largest margin between the separating hyperplane and the observations (Fig. 6.B). Points near the margins are called “support vectors” from which the classifier gets its name (Fig. 6.C). These data points are critical to fit the model to the dataset as they determine the position and the orientation of the hyperplane. Finally, how confidently we can say a point belongs to a group can be calculated based on the points distance from the margin. For example, we are more confident that Point 2 (Fig. 6.D, green circle) is correctly classified as a Mature mouse than Point 1 (Fig. 6.D, red circle) because it is a greater distance from line of division between the two groups.

#### Linear SVM Models of PC1 and PC2

Here, we create a C-type (binary classification) SVM model with the linear kernel, from the R packages e1071 [24] and kernlab [25] to create a SVM model of the PC1 and PC2 of the trabecular microCT outcome measures from the distal femur of Young and Mature mice. The SVM model classifies all of the Young and Mature mice correctly based on PC1 and PC2 of the trabecular microarchitecture (Table 5).

**Table 5:** Results of SVM model vs true groups of mission using distal femur trabecular microarchitecture PC1 and PC2.

Classified Groups		True Groups	
		*Mature*	*Young*
	*Mature*	30	0
	*Young*	0	36

#### Non-Linear SVM Models of PC1 and PC2

**Fig. 7:**
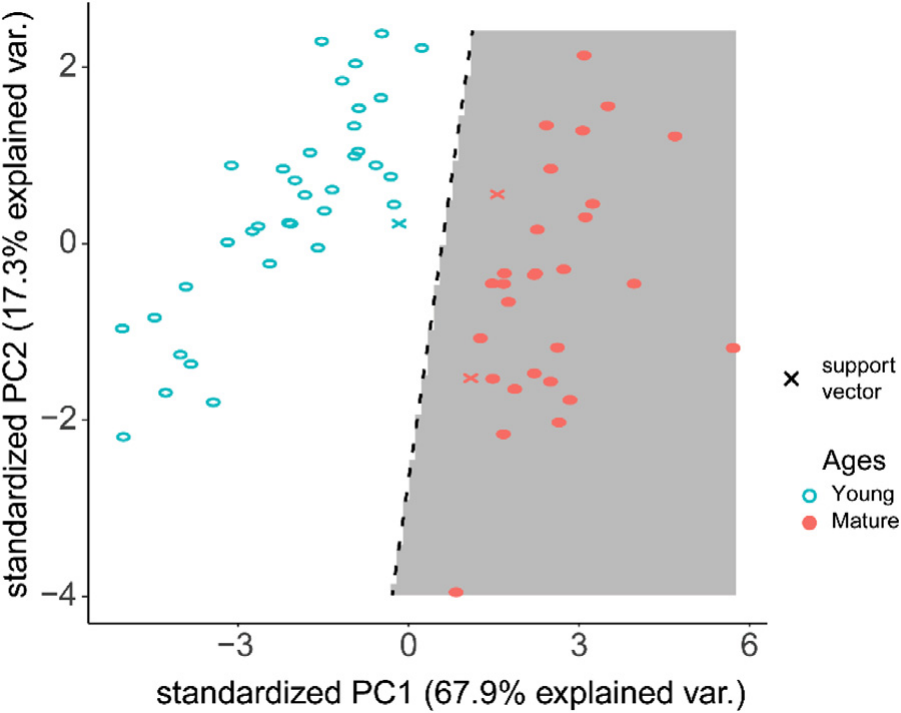
Linear SVM Model of PC1 and PC2 of Distal Femur Trabecular Microarchitecture outcomes. Open blue circles represent data from the Young population of the dataset, and closed red circles represent data points from Mature. White and gray areas represent the binarization of the data set, where the dashed line divides the two regions. *X* denotes a support vector.(For interpretation of the references to color in this figure legend, the reader is referred to the web version of this article.)

**Fig. 8:**
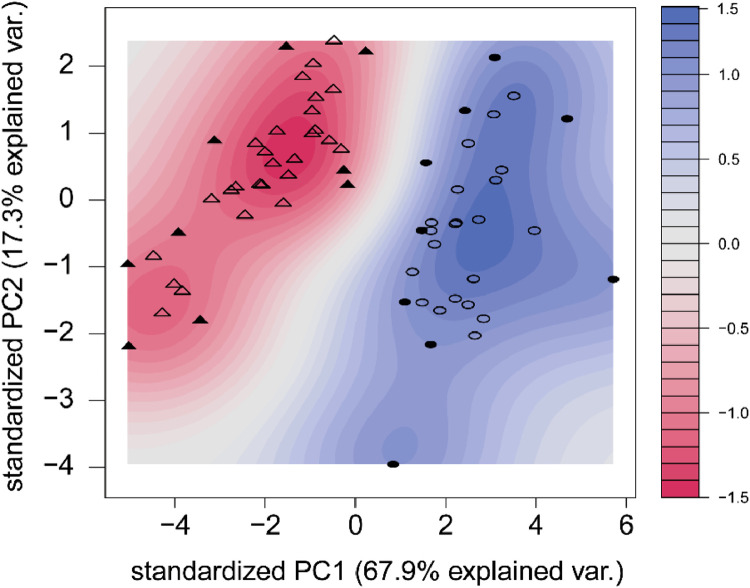
Non-linear SVM Model of PC1 and PC2 of Distal Femur Trabecular Microarchitecture outcomes. SVM classification plots of PC1 and PC2 by non-linear partitioning using a Polynomial kernel function from the R packages e1071 and kernlab. Circles represent data points from the Mature population of the dataset, and triangles from Young. Filled in circles or triangles denote a support vector. The color gradient indicates how confidently a new point would be classified based on its features.

While the classification of the dataset by age is easily partitioned by a linear fit SVM, when generalizing this classifier to more complicated classifications, groups may no longer be linearly separable. Due to the complex shape of the data, a non-linear partitioning kernel functions (Radial Basis, Polynomial, Laplacian, Bessel and Spline) can be used. However, non-linear kernels can make it more likely that to overfit the data. Therefore, we selected a polynomial kernel as a comparison to the linear kernel function for comparison (Fig. 7, Fig. 8).

#### Higher dimension SVM Models of PC1 and PC2

As with k-means analysis, for the accompanying dataset, we can evaluate how confidently a mouse could be classified into its treatment group (i.e. Young Baseline, Young Ground Control, Young Microgravity, Mature Baseline, Mature Ground Control, Mature Microgravity) given measures of trabecular microstructure or cortical structure. Conceptually, we investigated if the changes due to age and/or microgravity exposure generated differences in bone microarchitectural and structural properties that were uniquely identifiable. For example, we asked, “Is the bone microarchitectural and structural phenotype of Mature tibiae from microgravity mice distinct (i.e. confidently classifiably different) from normally loaded mice (e.g. Mature Baseline or Mature Ground Control) or from younger (e.g. Young microgravity) bone phenotypes?”

Visualizations of the SVM hyperplane are not easily available in higher dimensions. Rather, we track the efficacy of the classifications by their accuracy to the original divisions in the data (Table 6). Classification strength is calculated as the sum of the diagonal of the Table 6 (i.e., the total number of correctly classified samples) divided by the total number of samples.

**Table 6:** Results of SVM model vs True Groups of Age and Mission using Distal Femur Trabecular Microarchitecture PC1 and PC2. Classification Strength: 69.69%. Dark shaded cells (main diagonal of matrix) represent the correct classification, or agreement between the SVM Classified Groups and the True Groups (i.e., treatment groups from the study design). Light shaded cells indicate misclassification of the SVM model as compared to the True Groups.

Classified Groups		True Groups					
		Mature Baseline (*n* = 10)	Young Baseline (*n =* 12)	Mature Microgravity (*n =* 10)	Young Microgravity (*n =* 12)	Mature Ground Control (*n =* 10)	Young Ground Control (*n =* 12)
	Mature Baseline	6	0	0	0	5	0
	Young Baseline	0	9	0	2	0	1
	Mature Microgravity	0	0	9	0	3	0
	Young Microgravity	0	3	0	10	0	1
	Mature Ground Control	4	0	1	1	2	0
	Young Ground Control	2	0	0	6	0	10

With classification based on both age and microgravity exposure (i.e. Baseline, Ground Control, Microgravity), the SVM model most frequently misclassified Mature Baseline mice as Mature Ground Control. These findings are consistent with k-means, where these groups were highly overlapped. Notably, the overlapping of these two particular groups is not surprising as we anticipated little bone growth in normal gravity over 21 days in the Mature mice. By contrast, Young Baseline and Ground Control groups had the some of the highest proportion of correct classifications.

### Limitations and alternatives

We have demonstrated here how Principal Component Analysis, k-Means, and SVMs can be used for initial visual inspection of microCT data and to augment conventional group-wise comparison analyses. While k-means algorithms are best with large sample sizes, SVM models can struggle to define a line or function to separate more complex datasets that do not have a clear margin of separation. Furthermore, the k-means clustering algorithm works best for groups of roughly the same size, and is sensitive to outliers and variance [26]. Lastly, while not explored here, SVMs have become a popular tool for prediction. However, for predictive models, SVMs need a training data set to be effective, which is not always available. Clinical data may be a future area where SVM models are a more natural fit. For example, Sharma et. al. details a predictive SVM approach showing how micro-MRI trabecular bone microarchitecture data can be to identify Type 1 Gaucher disease [27]. This paper provided examples of “hard” clustering, where samples may only belong to one group. Alternative “soft” clustering approaches, such as a Gaussian Mixture model, samples can belong to multiple groups using weights or probabilities. Additionally, Bayesian k-means allow for assessments of likelihoods for clusterings [28].

Many of the results of this paper could have been accomplished solely through careful and robust non-parametric linear modelling. However, the visualizations could not have been done without dimensional reduction, and the consolidation of two groups in the k=5 analysis would have required a very low-power set of pairwise tests. Ultimately, we suggest these techniques not to replace parametric or non-parametric statistical testing, but as an additional means of demonstrating differences between microarchitectural outcomes.

## Declaration of Competing Interest

The authors declare that they have no known competing financial interests or personal relationships that could have appeared to influence the work reported in this paper.

## Appendix Supplementary materials

Image, application 1

Image, application 2

Image, application 3
